# RETRACTED: Alqahtani et al. Preparation and Characterization of Poly(vinyl acetate-co-2-hydroxyethyl methacrylate) and In Vitro Application as Contact Lens for Acyclovir Delivery. *Int. J. Mol. Sci.* 2023, *24*, 5483

**DOI:** 10.3390/ijms27177586

**Published:** 2026-08-25

**Authors:** Saad Mohammed Alqahtani, Rana Salem Al Khulaifi, Mohammed Alassaf, Waseem Sharaf Saeed, Idriss Bedja, Amal Aldarwesh, Abeer Aljubailah, Abdelhabib Semlali, Taieb Aouak

**Affiliations:** 1 Department of Chemistry, College of Science, King Saud University, Riyadh 11451, Saudi Arabia; salqahtani2@ksu.edu.sa (S.M.A.); ranaalkhulaifi@gmail.com (R.S.A.K.); alassafm926@gmail.com (M.A.); wsaeed@ksu.edu.sa (W.S.S.); akaljubailah@imamu.edu.sa (A.A.); 2 Department of Optometry, College of Applied Medical Sciences, King Saud University, Riyadh 11433, Saudi Arabia; bedja@ksu.edu.sa (I.B.); aaldarweesh@ksu.edu.sa (A.A.); 3 Groupe de Recherche en Écologie Buccale, Faculté de Médecine Dentaire, Université Laval, Quebec City, QC G1V 0A6, Canada; abdelhabib.semlali@greb.ulaval.ca

The journal retracts the article titled “Preparation and Characterization of Poly(vinyl acetate-co-2-hydroxyethyl methacrylate) and In Vitro Application as Contact Lens for Acyclovir Delivery” [1], cited above.

Following publication, concerns were brought to the attention of the Editorial Office regarding the integrity of Figure 12 presented in this article [1].

According to the journal’s standard procedures, the Editorial Office and Editorial Board conducted an investigation that identified image duplications between Figure 12 in this article [1] and micrographs presented in two previous publications [2,3], produced by similar authorship groups, reporting different experimental conditions.

Although the authors cooperated during the investigation, the explanations and supporting materials provided were not deemed sufficient by the Editorial Board to resolve the identified concerns. As a result, the Editorial Board has lost confidence in the reliability of the findings and has decided to retract this publication, as per MDPI’s retraction policy.

This retraction was approved by the Editor-in-Chief of the *International Journal of Molecular Sciences*.

The authors have been informed of this retraction. Saad Mohammed Alqahtani and Idriss Bedja have indicated their disagreement with the retraction, while Abdelhabib Semlali and Taieb Aouak provided no comments. The remaining authors have indicated their agreement with this decision.
